# Relationship preferences and experience of primary care patients in continuity of care: a case study in Beijing, China

**DOI:** 10.1186/s12913-017-2536-1

**Published:** 2017-08-22

**Authors:** Chaojie Liu, Yeqing Wu, Xueyang Chi

**Affiliations:** 1 School of Psychology and Public Health, La Trobe University, Melbourne, VIC 3086 Australia; 2 Fengtai Community Health Centre, Building 3, zone 2, Da Cheng Nan Li, Fengtai District, Beijing, 100040 China

**Keywords:** Continuity of care, Primary care, China

## Abstract

**Background:**

Continuity of care can bring a wide range of benefits to consumers, providers and health care systems. This study aimed to understand the relationship preferences of primary care patients and their associations with patient experience of continuity of care.

**Methods:**

A questionnaire survey was conducted on 700 patients who sought medical care from a community health organisation in Beijing. The survey contained four items examining the relationship preferences of the respondents, and a modified Questionnaire of Continuity between Care Levels (CCAENA) measuring patient experience of continuity of care based on a three dimensional (relational, informational and managerial) model. The associations between the relationship preferences and the experience of respondents in continuity of care was tested using a linear regression model controlling for age, sex, education, medical insurance, personal income and servicing facilities.

**Results:**

The respondents experienced relatively lower levels of informational and managerial continuity compared with relational continuity of care. More than 80% of respondents preferred free choice and a continuing relationship with doctors, compared with 59% who endorsed community facility control over hospital appointments. A preference for a continuing relationship with doctors was associated with all aspects of continuity of care. A preference in favour of community facility control over hospital appointments was a strong predictor of managerial continuity (*β* = 0.333, *p* < 0.001) and informational continuity (*β* = 0.256, *p* < 0.001). Patient preference for free choice of doctors was positively associated with relational continuity with specialists (*p* < 0.001), but not with primary care providers (*p* > 0.08). Perceived importance of information exchange was associated with relational and managerial continuity (*p* < 0.05), but not with informational continuity (*p* = 0.34).

**Conclusions:**

Patients prefer a high level of freedom of choice and sustained individual relationship with doctors. Relationship preferences of patients are associated with their experience of continuity of care. But patient strong preference for free choice of doctors is not aligned with relational continuity with primary care, a desirable feature of cost-effective healthcare systems.

**Electronic supplementary material:**

The online version of this article (doi:10.1186/s12913-017-2536-1) contains supplementary material, which is available to authorized users.

## Background

Continuity of care has been recognised as a fundamental building block for enhancing quality in health care [1, 2]. Continuity of care, especially in primary care, can bring a wide range of benefits to consumers, providers and health care systems, such as reduced hospital admissions, emergency visits and medical expenditure [3, 4]; better relationships and patient experience [5–7]; enhanced preventive care [8], and improved medication adherence, and safety and health care outcomes [9–11]. Warwick [12] argued that the core values of patient care (e.g. care, compassion, competence, communication, courage and commitment) can be better achieved in a system that emphasises continuity of care.

Continuity of primary care is needed because patient conditions have become increasingly complex, and in many cases need longitudinal care over an extended period of time or even life-long treatment; and having multiple providers may create a higher level of risk of miscommunication and mismanagement [1]. On the other hand, however, the most complex patients may benefit from seeing a diverse team of providers, simply because nowadays, health professionals have become highly subspecialised and patients’ needs can rarely be recognised and met by a single provider [13]. Unfortunately, medical subspecialisation has led to fragmented medical care, jeopardising coordination between care providers and across episodes of care [1]. When effective communication and coordination among providers are difficult to attain, a continuing interpersonal relationship with primary care providers is preferred [6, 9, 14]. de Jonge and colleagues [15] found that intra-partum referral from primary to secondary care makes patients feel unsafe. Having a predominant provider has been proved to be equally beneficial, whether it is with a primary care provider or with a specialist [3]. A recent study in the Netherlands demonstrated that poor continuity of primary care is associated with increased mortality of elderly patients [16].

The role of patients in continuity of care has started to attract attention in recent years. Unlike strategies for increasing care coordination, which have been traditionally anchored around provider-initiated actions (such as handover), continuity of care is a patient-oriented outcome. Over the past decade, patient/people centred care is gaining momentum, which calls for greater responsiveness to the values and principles held by patients. However, these values and principles may vary under different cultural contexts and remains elusive in terms of what patients want in relation to continuity of care. From the perspective of patients, care coordination is always desirable, but not necessary for continuity [1]. Although continuity of care does not equal coordinated care, a high level of patient-doctor relational continuity in primary care may result in better coordination of care [17].

Continuity of care is value-laden and heavily shaped by patient preference. This study aimed to investigate the relationship preferences of primary care patients, and their association with patient experience of continuity of care.

The study was undertaken in China. The Chinese health care system, like many other systems in the world, has evolved largely around the advancement of medical technologies. Professional, financial, and managerial arrangements are organised in a way that is tailored to the needs of a specific service or episode of care. Patient contribution to the design of the care process is limited. Some researchers believe that an improved information system, taking advantage of modern technology such as the Internet, can bring a solution to the poor coordination of care [18]. Sharing information not only improves communication, but also enables changes in the behaviours of health providers [19]. In reality, however, organisational barriers have created a significant challenge to such a proposal. Each organisation is an independent entity. In many cases, they are competing, instead of collaborating with each other. Furthermore, good continuity of care relies on close individual relationships between health providers and between health providers and consumers.

It is particularly important to understand how Chinese patients choose their preferred providers. Unlike in many developed systems, where referral arrangements promote the transference of information from one provider to another [20], China does not have an institutionalised referral system. This raises an important question about the role of patients in the continuity of their own care. Arguably, visits to health services are always irregular and episodic, and ongoing self-monitoring is important. In many outpatient clinics in China, medical records are kept by patients. Some studies found that patient-held information cards can promote continuity of care [19]. However, such an effect depends on the willingness and ability of the patient to maintain and share information.

This study examined patient preferences on two aspects of relationship with health providers: patient freedom of choice vs sustained provider-patient relationship (with restricted patient freedom); individual vs organisational-based provider-patient connections. The context of the current Chinese health system offered an ideal setting for exploring the association between patient relationship preferences and their correlations with patient experience in continuity of care. The findings of this study can also make a contribution to the international debate about whether the two concepts “continuity of care” and “patient-centred care” are harmonised.

## Methods

### Study setting

The study was conducted in a community health organisation in Beijing. Urban community health services are an initiative developed in the early 2000s by the Chinese government to break down hospital domination, a result of decades-long market-oriented reform [21]. In the 1980s, the Chinese government introduced market-oriented health reform. The share of the government budget for health expenditure reduced from 36.2% in 1980 to 15.5% in 2000 [22]. Health organisations were encouraged to grow by themselves through competition for consumers (with user payments). Patients did not have to be referred by a primary care provider to gain access to hospital care [21]. This put small and ill-equipped primary care facilities in a disadvantaged position. Patients have an overwhelming belief that well-equipped large hospitals can provide higher quality of care compared with their primary care counterparts. As a result, the hospital share of outpatient visits increased from 41% in 1980 to 61% in 2000 [22]. But meanwhile, the costs of care surged and continuity and coordination of care suffered. Although the government has increased its investment in primary care over the past decade, a patient referral system is still absent. According to the most recent report from the National Health and Family Planning Commission, 340,000 urban community health centres/stations (2.4 per ten thousand population) had been established by the end of June 2016. However, hospital share in outpatient care remains high (40.8%) [22].

The participating organisation in this study is the only public community health facility providing primary care and public health services to 160,000 people across a geographic area of 12 km^2^. About 77% of the covered population entered into a voluntary contract with the organisation, although they were not obligated to choose this organisation as a first contact point or as an exclusive primary care provider. The organisation has one centre (78 staff) and five outreach stations (8–32 staff). There are 165 employees, with doctors, nurses and pharmacists accounting for 44, 26 and 19% of the workforce, respectively. They provide general practice consultations, management of chronic conditions (including psychiatric conditions), health education, maternal and child health care, vaccinations, control of infectious disease, and supportive services for public health agencies. The outpatient clinics receive an average of 1000 patient visits a day. About 70% of patients are aged between 55 and 64 years (32%) or ≥65 years (38%); 90% were covered by the urban insurance scheme.

### Participants and data collection

A questionnaire survey was undertaken. Data were collected over a 10-day period (9–18 June 2014). Patients visiting the participating organisation during this period of time were invited to participate in this survey. Those who were younger than 18 years and those who were deemed too sick or too frail as assessed by the doctors were excluded from the study. The eligible patients were advised to contact the researchers after completion of their services, should they volunteer to participate in this study.

The questionnaire was administered through face-to-face interviews. The interviewers did not have a servicing relationship with the participants. Written informed consent was obtained before the commencement of the interviews. A total of 700 questionnaires were completed, representing about 10% of eligible patients attending the participating organisation during the study period. A sample size of 400 would provide 90% power to detect a 10% difference in continuity of care (CoC) scores between the participants with different relationship preferences (based on a 5% significance level, a standard deviation of 1.02 for CoC scores, and a ratio of 1:9 between those who held different preferences). We increased the sample size to 700 to enable reliable factor analysis (with four factors containing 20 variables) [23] and regression modelling (with 18 independent variables) [24].

### Measurement

The concept of continuity was defined as the patient experience of care over time in terms of coherence, connectedness and unbrokenness of care [25]. Several instruments are available for measuring CoC. Although CoC can be measured from the provider’s perspective (e.g. proportion of regular patients in all visits) [2], the majority measure CoC from the patient’s perspective. For example, the Bice-Boxerman CoC index measures dispersion (the number of different providers seen) [10]; Usual Provider of Continuity (UPC) measures density (number of visits with the same provider) [26]; Modified Continuity Index (MCI) and Modified Modified Continuity Index (MMCI) measure concentration of care with providers at the population and individual levels, respectively [6, 26]; and Sequential Continuity Index (SECON) measures sequential patterns of patient visits [27]. These indices are easy to gather, but have been subject to increasing criticisms recently. Donaldson [28] labelled them as convenient indices and argued that these indices fail to take into account the contents of the visits and they do not tell whether these visits have been connected and whether the goals and efforts of the service providers are well aligned with the goals of the patients. Incomplete CoC measurements may have resulted in an underestimation of the link between improved CoC and health outcomes [29]. A review of the qualitative studies on patient perceptions shows that patients, especially those with long-term conditions, often consider that several providers know them well and CoC would not be disrupted by maintaining multiple patient-provider relationships [2].

There has been a growing consensus on the multidimensional nature of CoC over the past decade. The most frequently used CoC model is a three-dimensional one, covering relational, informational and managerial continuity of care [2, 11, 25, 26, 30, 31]. This model addresses the needs of patients and concerns about the quality of patient-provider relationships. Relational continuity refers to the familiarity between a patient and his/her providers. Informational continuity indicates the availability and use of full information (including information obtained from past and from others) relating to the patient served by a care provider. Managerial continuity measures the consistency of care across different types (interdisciplinary), sites (geographical), and episodes of care (chronological), as well as responsiveness to the changing circumstances of the patients.

We chose to use a modified Questionnaire of Continuity between Care Levels (CCAENA) to measure patient experience of CoC based on the three-dimensional model [31, 32]. The original questionnaire contains seven items measuring relational continuity with primary care and specialist care, respectively; four items measuring informational continuity and three items measuring managerial continuity. One of the informational continuity items asked “After seeing the specialist my GP discusses the visit with me”, which was deemed irrelevant under the Chinese context and was withdrawn from the questionnaire (Additional file 1: questionnaire). The CCAENA produced high internal consistency in this study sample, with Cronbach’s Alpha 0.934, 0.915, 0.900, 0.892, and 0.770 for the overall questionnaire and its four CoC domains, respectively. Confirmatory Factor Analysis (CFA) proved a good fit of model [30] based on standard indices (GFI = 0.912; CFI = 0.943; NFI = 0.927; TLI = 0.932; RMSEA = 0.069 (90% CI 0.063, 0.074); χ^2^ = 683.358 (159) *p* < 0.001) (Additional file 2: CFA results).

We designed four items, examining the preference of respondents with choice and relationships. We asked the participants: whether they believed “freedom of choice is more important than receipt of facility coordinated care”; whether “patient information should be shared between community and hospital facilities”; whether “hospital appointments should be made by community facilities”; and whether “the patient-doctor relationship is more important than the patient-facility relationship”. These questions were designed to address the concerns of health service managers: how to maintain a balance between patient freedom of choice and organisational control over patient care within and across facilities. It is important to note that doctors in China are full-time employees and inter-facility patient transfer is usually made through organisational arrangements rather than individual connections between doctors [33].

### Data analysis

Data analyses were performed using the SPSS 22.0. The relationship preferences were measured on a five-point Likert scale (from totally disagree to totally agree). We used frequency distributions to describe the preferences of the respondents and Spearman correlations to describe the correlations between the four aspects of preferences. We then recoded the preference variables into dichotomous measurements (0 = “disagree” including “totally disagree, disagree, and not sure”, 1 = “agree” including “agree and totally agree”) and explored the associations between the preference measurements and the characteristics of the respondents (age, sex, education, medical insurance, personal income, and service facilities) using a logistic regression model.

An average aggregated score for each of the CoC experience domains (summed item scores divided by the number of items) was calculated. We examined the differences of the CoC scores of the respondents who had different relationship preferences using ANOVA analyses. We then developed linear regression models, with the four CoC experience domains serving as dependent variables. The associations between the four preference measurements (as independent variables) and the CoC experience of the respondents were tested after controlling for age, sex, education, medical insurance, personal income and setting. The control variables were entered into the models first before the preference measurements were introduced into the models (hierarchical approach). The regression models adopted maximum likelihood estimations, with an enter/exit criterion (α) of 0.05/0.10.

### Ethics approval

Ethics approval was obtained from the Faculty Human Ethics Committee of La Trobe University (FHEC09/246) and Fengtai Community Health Centre of Beijing.

## Results

### Characteristics of respondents

About 59% of respondents were women; 61% of respondents were aged between 45 and 64 years old. Almost half the participants had not attended tertiary education. The majority (87%) were covered by the urban social health insurance schemes. The monthly income of the participants was relatively low, with over 80% falling into the middle and low range of 2001–6000 RMB (equivalent to US$320–960) (Table 1).

**Table 1:** Characteristics of study participants

Characteristic		Number of respondents	Percentage	Population of users in 2014
Sex				
	Female	414	59.1	62.2
	Male	286	40.9	37.8
Age				
	<35	50	7.1	7.6
	35–44	93	13.3	6.3
	45–54	196	28.0	16.0
	55–64	229	32.7	32.3
	≥65	132	18.9	37.8
Education				
	Without a degree	347	49.6	
	Associate degree	199	28.4	
	Bachelor degree	137	19.6	
	Postgraduate degree	17	2.4	
Medical insurance				
	No insurance	13	1.9	8.3
	Rural insurance	40	5.7	0.9
	Urban insurance	610	87.1	90.0
	Free medicine	32	4.6	0.8
	Commercial insurance	5	0.7	0
Monthly income				
	≤2000	74	10.6	
	2001–4000	397	56.7	
	4001–6000	167	23.9	
	6001–8000	45	6.4	
	8001–10,000	14	2.0	
	>10,000	3	0.4	
Setting				
	Centre	350	50.0	
	Station	350	50.0	

### Relationship preferences

The respondents tended to endorse the concept of continuity of care tested in this study. Almost 90% agreed (63% totally agreed) that information needs to be shared across community and hospital facilities. However, there was overwhelming agreement on patient freedom of choice. About 87% of respondents favoured patient freedom of choice over restricted choice facilitated through community health facilities. More than 41% of respondents did not embrace community facility control over hospital appointments. The majority (82%) preferred a continuing individual relationship with doctors instead of a continuing patient-facility relationship (Table 2).

**Table 2:** Relationship preferences of respondents

Rating	It is important to share information between community and hospital facilities		Hospital appointments must be arranged by community facilities		It is more important to have access to the same doctor (or a team) than to the same facility (hospital)		It is more important to have freedom of choice than to have access to coordinated services through a health facility	
	Number of respondents	Percentage	Number of respondents	Percentage	Number of respondents	Percentage	Number of respondents	Percentage
Totally disagree	18	2.6	112	16.0	11	1.6	10	1.4
Disagree	12	1.7	70	10.0	22	3.1	16	2.3
Not sure	47	6.7	106	15.1	94	13.4	68	9.7
Agree	182	26.0	154	22.0	232	33.1	216	30.9
Totally agree	441	63.0	258	36.9	341	48.7	390	55.7
Total	700	100.0	700	100.0	700	100.0	700	100.0

The four preference measurements were moderately correlated, with a Spearman correlation coefficient ranging from 0.22 to 0.57 (Table 3). The highest correlation was found between “free choice of doctors” and “continuing patient-doctor relationship” (*r* = 0.57, *p* < 0.001).

**Table 3:** Spearman Correlations (r ± SE) between relationship preferences of respondents

	It is important to share information between community and hospital facilities (Q1)	Hospital appointments must be arranged by community facilities (Q2)	It is more important to have access to the same doctor (or a team) than to the same facility (hospital) (Q3)	It is more important to have freedom of choice than to have access to coordinated services through a health facility (Q4)
Q1	1			
Q2	0.234 ± 0.036*	1		
Q3	0.241 ± 0.038*	0.397 ± 0.034*	1	
Q4	0.215 ± 0.038*	0.238 ± 0.036*	0.569 ± 0.032*	1

*p < 0.001

The relationship preferences of patients were associated with their gender, income, insurance and servicing facilities (Table 4). Women were more likely to endorse a continuing patient-doctor relationship than their male counterparts (OR = 1.579, *p* = 0.036). The respondents with lower incomes were more likely to agree with the importance of “community facility control over hospital appointments”, “continuing patient-doctor relationship”, and “free choice of doctors” compared with those with a higher level of income. The respondents covered by the urban social health insurance were more likely to endorse information exchange (OR = 3.873, *p* = 0.024) and a continuing doctor-patient relationship (OR = 3.890, *p* = 0.025). The respondents seeking services from the centre were more likely to choose “community facility control over hospital appointments” (OR = 1.937, *p* < 0.001) and a “continuing patient-doctor relationship” (OR = 3.151, *p* < 0.001), but less likely to endorse “information exchange” (OR = 0.514, *p* = 0.013) compared with those who sought services from the outreach stations.

**Table 4:** Logistic regression models on relationship preferences (1 = agreed, 0 = disagreed/not sure) of patients

Characteristics		Cross-facility information exchange				Community facility control over hospital appointments				Continuing patient-doctor relationship				Free choice of doctors			
		OR	95% Confidence Interval		*p*	OR	95% Confidence Interval		*p*	OR	95% Confidence Interval		*p*	OR	95% Confidence Interval		*p*
Sex	Women	.939	.566	1.561	.809	.951	.686	1.318	.761	**1.579**	**1.031**	**2.419**	**.036**	.962	.604	1.533	.871
Age																	
	Age < 35	1.433	.443	4.629	.548	1.050	.495	2.228	.898	.382	.146	1.002	.050	.777	.295	2.049	.610
	Age 35–44	2.647	.884	7.922	.082	1.254	.665	2.363	.485	.902	.377	2.157	.816	1.383	.560	3.414	.482
	Age 45–54	1.200	.575	2.505	.626	.957	.583	1.572	.863	.905	.447	1.831	.781	1.166	.581	2.341	.666
	Age 55–64	1.351	.672	2.712	.398	1.179	.741	1.876	.487	.550	.296	1.022	.059	1.372	.709	2.654	.348
Monthly income																	
	<2000	.541	.055	5.289	.597	**4.161**	**1.235**	**14.026**	**.021**	3.535	.879	14.219	.075	**4.908**	**1.162**	**20.740**	**.030**
	2001–4000	.629	.073	5.447	.673	2.737	.948	7.906	.063	**3.813**	**1.198**	**12.134**	**.023**	**3.379**	**1.062**	**10.749**	**.039**
	4001–6000	.418	.049	3.527	.423	2.026	.706	5.815	.189	**3.716**	**1.172**	**11.784**	**.026**	**3.259**	**1.034**	**10.271**	**.044**
	6001–8000	.260	.028	2.377	.233	.942	.295	3.005	.920	.787	.235	2.638	.698	2.161	.602	7.753	.237
Education	No college qualification	1.151	.594	2.230	.677	1.279	.855	1.914	.231	1.401	.803	2.443	.235	1.116	.617	2.017	.717
Insurance																	
	Rural insurance	2.405	.642	9.018	.193	1.633	.511	5.218	.408	1.813	.488	6.735	.374	2.620	.626	10.957	.187
	Urban insurance	**3.873**	**1.199**	**12.514**	**.024**	2.132	.755	6.022	.153	**3.890**	**1.191**	**12.709**	**.025**	3.113	.929	10.428	.066
	Free medicine	2.327	.499	10.853	.282	1.958	.539	7.116	.308	2.527	.562	11.358	.227	2.157	.468	9.944	.324
Facility	Centre	**.514**	**.304**	**.870**	**.013**	**1.937**	**1.401**	**2.678**	**.000**	**3.151**	**2.005**	**4.950**	**.000**	1.066	.673	1.687	.787
Constant		4.823			.204	.177			.021	.231			.077	.551			.478

Bold: *p* < 0.05

### Experience of continuity of care and its association with relationship preferences

The participants gave lower scores (paired *t* tests, *p* < 0.001) to informational (CoC scores = 3.98 ± 1.02) and managerial (4.07 ± 0.84) continuity compared with relational continuity (4.60 ± 0.56 with primary care and 4.35 ± 0.65 with specialist care).

**Fig. 1:**
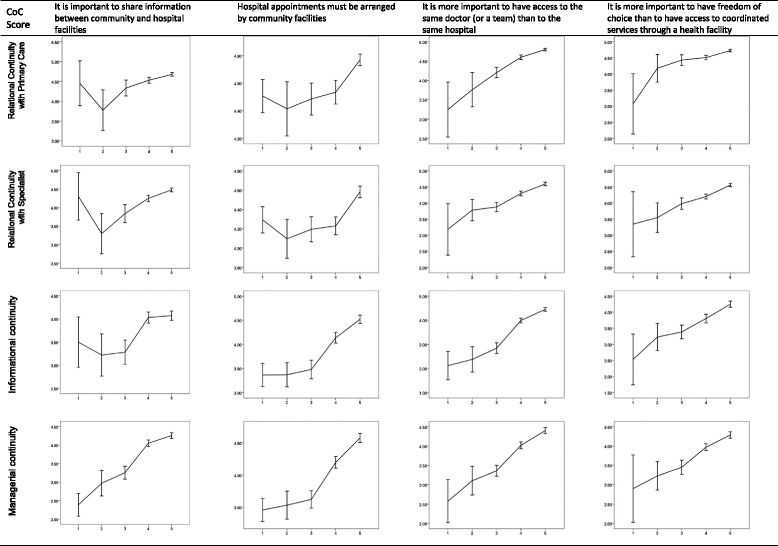
Continuity of Care (CoC) scores (Mean ± 95% CI) of respondents with different relationship preferences, ranging from totoally disagree (1) to totoally agree (5)

Statistical differences in CoC scores were found between those with different relationship preferences (Fig. 1). The linear regression models revealed that all of the four preference measurements were associated with managerial continuity; while two or three preference measurements were associated with informational continuity and relational continuity (Table 5). A preference for a continuing patient-doctor relationship was the strongest predictor of relational (β = 0.409 for primary care; 0.287 for specialist care) and informational (β = 0.439) continuity; whereas, a preference of “community facility control over hospital appointments” was the strongest predictor of managerial continuity (β = 0.333). The perceived importance of information exchange was associated with relational continuity and managerial continuity, but not with informational continuity. By contrast, a preference of “community facility control over hospital appointments” was positively associated with informational continuity. Patient preference of “free choice of doctors” was positively associated with relational continuity with specialists (*p* < 0.001), but not with primary care providers (*p* > 0.08).

**Table 5:** Linear regression models on patient experience in continuity of care

		Relational Continuity Primary Care				Relational Continuity Specialist				Informational Continuity				Managerial Continuity			
		B	Std. Error	Standardised β	*p*	B	Std. Error	Standardised β	*p*	B	Std. Error	Standardised β	*p*	B	Std. Error	Standardised β	*p*
Relationship preferences																	
	Information exchange	.135	.064	.076	**.036**	.328	.076	.159	**.000**	.100	.105	.031	.340	.755	.085	.281	**.000**
	Community facility control over hospital appointments	−.035	.043	−.031	.411	−.048	.050	−.037	.341	.533	.070	.256	**.000**	.568	.056	.333	**.000**
	Continuing patient-doctor relationship	.592	.058	.409	**.000**	.481	.069	.287	**.000**	1.165	.096	.439	**.000**	.399	.077	.183	**.000**
	Free choice of doctors	.103	.060	.063	.086	.301	.071	.159	**.000**	.084	.098	.028	.391	.299	.079	.121	**.000**
Sex (Female = 1)		−.006	.039	−.005	.873	−.040	.046	−.031	.385	−.005	.063	−.003	.934	.034	.051	.020	.513
Age 35–44		.089	.086	.054	.301	−.028	.102	−.015	.786	.157	.140	.052	.263	.036	.114	.015	.750
Age 45–54		.084	.079	.068	.286	−.071	.094	−.049	.448	.184	.129	.081	.153	.106	.104	.057	.309
Age 55–64		.087	.080	.073	.277	−.056	.095	−.041	.555	−.003	.131	−.001	.982	.000	.106	.000	.997
Age 65 and over		.080	.089	.056	.367	−.044	.106	−.027	.675	.000	.146	.000	.999	.002	.118	.001	.985
Without college education		.067	.048	.060	.160	.044	.057	.034	.440	.064	.078	.031	.409	−.005	.063	−.003	.933
Rural health Insurance		−.104	.139	−.043	.457	.272	.165	.098	.100	−.057	.228	−.013	.801	.103	.184	.028	.577
Urban health Insurance		.075	.125	.045	.551	.390	.148	.203	**.009**	.007	.204	.002	.972	−.045	.165	−.018	.784
Free medicine		.024	.154	.009	.878	.390	.183	.126	**.033**	.023	.252	.005	.928	.064	.204	.016	.754
Income ≤2000 Yuan		.022	.144	.012	.881	.221	.171	.105	.198	.106	.236	.032	.653	.091	.191	.033	.634
Income 2001–4000 Yuan		.101	.128	.090	.427	.150	.152	.116	.321	.086	.209	.042	.681	.057	.169	.033	.737
Income 4001–6000 Yuan		.140	.127	.107	.272	.129	.151	.085	.395	.024	.208	.010	.910	.080	.168	.041	.635
Income 6001–8000 Yuan		−.187	.139	−.082	.181	−.037	.165	−.014	.821	−.227	.228	−.054	.320	−.007	.184	−.002	.971
Setting (Centre = 1)		−.031	.040	−.028	.435	.051	.047	.040	.279	.087	.065	.043	.181	.083	.053	.050	.115
Constant		3.690	.187		.000	2.949	.222		.000	2.351	.306		.000	2.350	.248		.000
	R^2^	.265				.228				.418				.435			

Bold: *p* < 0.05

## Discussion

The study participants experienced a higher level of relational continuity than informational continuity, similar to studies undertaken elsewhere by Aller et al. [34]. But this finding is different from a Spanish study, which revealed more relational problems than information transference [7].

Patient preferences for a personal choice and individualised relationship with doctors are predictors of relational continuity. However, we found that patients who preferred “free choice of doctors” experienced better relational continuity with specialists, but not with primary care providers. A randomised controlled trial demonstrated that the usual care model under the patient’s own initiative produced the highest UPC score [35]. Kao et al. also found that patients who have enough choice of providers are more likely to trust their physician [36]. The lack of association between “free choice” and “relational continuity with primary care providers” may be an indication of a lack of confidence of the Chinese patients in primary care. The quality of primary care is often deemed low in China [21]. The shortage of individual-based communication and referral relationships between primary care doctors and specialists in China may further discourage a continuing relationship between patients and primary care doctors [33]. For patients, there is always a trade-off between continuity and access [37, 38]. But it is unreasonable to assume that patients prefer not to stick with one or a small team of providers when they enjoy freedom of choice [2]. Patients are more likely to choose to maintain a continuing relationship with a care provider whom they believe is able to deliver high quality consultations (e.g. attentiveness, inspiration of confidence, medical knowledge etc.) [2].

The Chinese government encourages organisational arrangements for inter-facility patient transfer despite a lack of individual referral between doctors [21]. An organisational-based approach is believed to have a strong capability to meet the need for continuity of care [37]. This is because two or more care providers can work together which makes them easily accessible at the time when patients need them. This may be true and team-based contracting is indeed gaining momentum in the current Chinese primary care reform. But the findings of this study clearly indicate that patients prefer a continuing individual relationship between patients and doctors instead of a continuing patient-facility relationship. Although a preference for “community facility control over hospital appointments” is a strong predictor of informational continuity, it is not associated with relational continuity. From the patient’s point of view, facility-dependent may improve the quantity of the continuing relationship, but not necessarily the quality of the relational continuity.

Despite a higher level of relational continuity, informational continuity at the primary-specialist care interface was found to be relatively low in this study. A lack of informational continuity is a serious issue of concern. Even in a system with almost complete transference of information, medical errors still occur. A US study revealed that medication discrepancy between primary care and hospital care can be as large as more than 30%, even when information transference is secured for almost all of the cases [20].

Interestingly, patient-perceived importance of “information exchange” is not associated with informational continuity. In addition, those who preferred “free choice of doctors” did not experience a higher level of informational continuity either. In contrast, the patients who preferred a provider-initiated approach (such as hospital appointments) tended to rate their experience of informational continuity higher. This may be an indication of patients feeling a lack of control and influence over information continuity. Uijen and colleagues argued that services initiated and coordinated by a care provider may enable a more frequent review of patient conditions, without necessarily jeopardising connectedness of care [35].

All of the four aspects of relationship preferences of patients were found to be associated with managerial continuity. However, it is important to note that patient influence on managerial continuity may be limited because managerial continuity is subject to the heavy influence of organisational policies and procedures, in which patients’ participation is often limited. Financial and organisational policies can sometimes also jeopardise the ability of patients to maintain relational continuity. In this study, we found that the urban social health insurance and free medicine scheme are negatively associated with relational continuity with specialists. A previous study also found that the technological-oriented service model and an emphasis on productivity can undermine continuity of care [28].

This study was conducted in one primary care setting. Attempts to generalise the findings of this study must be cautious. No causal inferences should be made, given the nature of the cross-sectional design of this study. The data of this study were collected through face-to-face interviews, which may also encourage the participants to respond in a socially desirable way leading to a bias toward better experience. However, the selection and responsive bias is less likely to influence the findings about the factors associated with continuity of care.

The instrument used in this study has advantages over those that bias towards counts of numbers of continuing relationships. But like almost all other available instruments, it still puts a heavier weight on relational continuity than managerial continuity [25]. Further development of continuity of care measurements is warranted.

## Conclusions

Patients prefer a high level of freedom of choice and sustained individual relationship with doctors. However, strong patient preference for free choice of doctors is not aligned with a strong continuing relationship with primary care, a critical feature of better and more cost-efficient healthcare systems [16].

While relational continuity is important, increased attention should be paid to informational and managerial continuity in China. This is not only because informational continuity was found to be low in this study, but also because managerial and informational continuity are more likely than relational continuity to impose a direct impact on patient care outcomes [39]. There is evidence showing that patients may be willing to sacrifice relational continuity when they perceive a low impact from disrupted relational continuity [40]. Meanwhile, however, service providers should be encouraged to develop innovative approaches to care delivery, enhancing patient-doctor interactions [41]. It is important to maintain a balance between sustained patient-provider relationships and freedom of choice. While sustained patient-provider relationships facilitate information exchange, patient freedom of choice may offer patients bargaining power to ensure their goals have been respected and integrated into the efforts of providers [28].

## Additional files

Additional file 1: Appendix 1. Questionnaire. (DOCX 24 kb)

Additional file 2: Appendix 2. CFA Results. (DOCX 135 kb)

## Abbreviations

CCAENA: Questionnaire of Continuity between Care Levels
CoC: Continuity of Care
MCI: Modified Continuity Index
MMCI: Modified Modified Continuity Index
OR: Odds Ratio
SECON: Sequential Continuity Index
UPC: Usual Provider of Continuity

## References

[1] Chen LM, Ayanian JZ (2014). Care continuity and care coordination: what counts?. JAMA Intern Med.

[2] Bjorkelund C, Maun A, Murante AM, Hoffman K, De Maeseneer J, Farkas-Pall Z (2013). Impact of continuity on quality of primary care: from the perspective of citizens’ preferences and multimorbidity - position paper of the European forum for primary care. Qual Prim Care.

[3] Romaire MA, Haber SG, Wensky SG, McCall N (2014). Primary care and specialty providers: an assessment of continuity of care, utilization, and expenditures. Med Care.

[4] King M, Jones L, Richardson A, Murad S, Irving A, Aslett H, Ramsay A, Coelho H, Andreou P, Tookman A (2008). The relationship between patients’ experiences of continuity of cancer care and health outcomes: a mixed methods study. Br J Cancer.

[5] Williams J (2014). Potential benefits of relationship continuity in patient care. Br J Nurs.

[6] Katz DA, McCoy K, Sarrazin MV (2014). Does improved continuity of primary care affect clinician-patient communication in VA?. J Gen Intern Med.

[7] Medina-Mirapeix F, Oliveira-Sousa SL, Sobral-Ferreira M, Montilla-Herrador J, Jimeno-Serrano FJ, Escolar-Reina P (2013). What elements of the informational, management, and relational continuity are associated with patient satisfaction with rehabilitation care and global rating change?. Arch Phys Med Rehabil.

[8] Menec VH, Sirski M, Attawar D (2005). Does continuity of care matter in a universally insured population?. Health Serv Res.

[9] Hong JS, Kang HC (2014). Relationship between continuity of ambulatory care and medication adherence in adult patients with type 2 diabetes in Korea: a longitudinal analysis. Med Care.

[10] Leleu H, Minvielle E (2013). Relationship between longitudinal continuity of primary care and likelihood of death: analysis of national insurance data. PLoS One.

[11] Chen CC, Tseng CH, Cheng SH (2013). Continuity of care, medication adherence, and health care outcomes among patients with newly diagnosed type 2 diabetes: a longitudinal analysis. Med Care.

[12] Warwick C (2014). Another ‘C’ is needed: continuity. Nurs Manag (Lond).

[13] Gulliford M, Naithani S, Morgan M (2006). What is ‘continuity of care’?. J Health Serv Res Policy.

[14] Gjevjon ER, Eika KH, Romoren TI, Landmark BF (2014). Measuring interpersonal continuity in high-frequency home healthcare services. J Adv Nurs.

[15] de Jonge A, Stuijt R, Eijke I, Westerman MJ (2014). Continuity of care: what matters to women when they are referred from primary to secondary care during labour? A qualitative interview study in the Netherlands. BMC Pregnancy Childbirth.

[16] Maarsingh OR, Henry Y, van de Ven PM, Deeg DJ (2016). Continuity of care in primary care and association with survival in older people: a 17-year prospective cohort study. Br J Gen Pract.

[17] O’Malley AS, Cunningham PJ (2009). Patient experiences with coordination of care: the benefit of continuity and primary care physician as referral source. J Gen Intern Med.

[18] Dickerson AE, Sensmeier J (2010). Sharing data to ensure continuity of care. Nurs Manag.

[19] McBride A, Burey L, Megahed M, Feldman C, Deaton C. The role of patient-held alert cards in prom1oting continuity of care for heart failure patients. Eur J Cardiovasc Nurs. 2014;13(1):71–7. 10.1177/1474515113478531 23406674

[20] McMillan A, Trompeter J, Havrda D, Fox J (2013). Continuity of care between family practice physicians and hospitalist services. J Healthc Qual.

[21] Liu C (2003). Closing the gap between policy and reality: a study of community health services in Chengdu and Panzhihua.

[22] National Health and Family Planning Commission (2016). Health statistical reports.

[23] Wolf EJ, Harrington KM, Clark SL, Miller MW (2013). Sample size requirements for structural equation models: an evaluation of power, bias, and solution propriety. Educ Psychol Meas.

[24] Dupont WD, Plummer WD (1998). Power and sample size calculations for studies involving linear regression. Control Clin Trials.

[25] Waibel S, Henao D, Aller MB, Vargas I, Vazquez ML (2012). What do we know about patients’ perceptions of continuity of care? A meta-synthesis of qualitative studies. Int J Qual Health Care.

[26] Health Quality O (2013). Continuity of care to optimize chronic disease management in the community setting: an evidence-based analysis. Ont Health Technol Assess Ser.

[27] Uijen AA, Schers HJ, Schellevis FG, van den Bosch WJ (2012). How unique is continuity of care? A review of continuity and related concepts. Fam Pract.

[28] Donaldson MS (2001). Continuity of care: a reconceptualization. Med Care Res Rev.

[29] Wei X, Barnsley J, Zakus D, Cockerill R, Glazier R, Sun X (2008). Assessing continuity of care in a community diabetes program: initial questionnaire development and validation. J Clin Epidemiol.

[30] Bentler SE, Morgan RO, Virnig BA, Wolinsky FD (2014). Evaluation of a patient-reported continuity of care model for older adults. Qual Life Res.

[31] Aller MB, Vargas I, Garcia I, Coderch J, Colomés L, Llopart JR, Ferran M, Sánchez-Pérez I, Vázquez ML (2013). A tool for assessing continuity of care across care levels: an extended psychometric validation of the CCAENA questionnaire. Int J Environ Res Public Health.

[32] Aller MB, Colome JM, Waibel S, Vargas I, Vazquez ML (2013). A first approach to differences in continuity of care perceived by immigrants and natives in the Catalan public healthcare system. Int J Environ Res Public Health.

[33] Liu C, Bartram T, Casimir G, Leggat SG (2015). The link between participation in management decision-making and quality of patient care as perceived by Chinese doctors. Public Manag Rev.

[34] Aller MB, Vargas I, Waibel S, Coderch J, Sanchez-Perez I, Colomes L, Llopart JR, Ferran M, Vazquez ML (2013). A comprehensive analysis of patients’ perceptions of continuity of care and their associated factors. Int J Qual Health Care.

[35] Uijen AA, Bischoff EW, Schellevis FG, Bor HH, van den Bosch WJ, Schers HJ (2012). Continuity in different care modes and its relationship to quality of life: a randomised controlled trial in patients with COPD. Br J Gen Pract.

[36] Kao AC, Green DC, Davis NA, Koplan JP, Cleary PD (1998). Patients’ trust in their physicians: effects of choice, continuity, and payment method. J Gen Intern Med.

[37] Gupta R, Bodenheimer T (2013). How primary care practices can improve continuity of care. JAMA Intern Med.

[38] Haggerty JL, Reid RJ, Freeman GK, Starfield BH, Adair CE, McKendry R (2003). Continuity of care: a multidisciplinary review. BMJ.

[39] van Servellen G, Fongwa M, Mockus D’Errico E (2006). Continuity of care and quality care outcomes for people experiencing chronic conditions: a literature review. Nurs Health Sci.

[40] Turner D, Tarrant C, Windridge K, Bryan S, Boulton M, Freeman G, Baker R (2007). Do patients value continuity of care in general practice? An investigation using stated preference discrete choice experiments. J Health Serv Res Policy.

[41] Wei X, Barnsley J, Zakus D, Cockerill R, Glazier R, Sun X (2008). Evaluation of a diabetes management program in China demonstrated association of improved continuity of care with clinical outcomes. J Clin Epidemiol.

