# Supplementary material for: Relationship preferences and experience of primary care patients in continuity of care: a case study in Beijing, China
Source: BMC Health Serv Res. 2017 Aug 22;17:585. doi: 10.1186/s12913-017-2536-1 (PMC5568350; doi:10.1186/s12913-017-2536-1)
Supplement: Supplementary file 1 — Questionnaire. (DOCX 24 kb) [file 12913_2017_2536_MOESM1_ESM.docx]

问卷编号： ________

消费者角度连续性医疗卫生服务调查问卷（ NCQ ）

**说明**：设计这些问题是为了解您对连续性医疗卫生服务的看法，请您自己表达您对这些问题的真实想法。您的看法可能对以后的相关政策有积极的影响。

请认真阅读每一个问题，并在1和5之间的数字选择一个来表示您的观点，其对应关系为1（非常不同意）、2（有点不同意）、3（不能决定是否同意或不同意）、4（部分同意）或5 （非常同意）。

| 对于问题您的赞成度 | 非常不同意 | 有点不同意 | 不确定同意或不同意 | 部分同意 | 非常同意 |
| --- | --- | --- | --- | --- | --- |
| 社区医院全科医生方面： | | | | | |
| 1. 您对您的社区全科医生的专业能力很自信。 | 1 | 2 | 3 | 4 | 5 |
| 1. 您相信您的社区全科医生给您提供的服务。 | 1 | 2 | 3 | 4 | 5 |
| 1. 您乐意与您的社区全科医生讨论关于您的疑虑和健康问题。 | 1 | 2 | 3 | 4 | 5 |
| 1. 您的社区全科医生理解您对他/她就您的健康问题的表述。 | 1 | 2 | 3 | 4 | 5 |
| 1. 您的社区全科医生给予您的信息容易理解。 | 1 | 2 | 3 | 4 | 5 |
| 1. 您的社区全科医生给予您的信息是有效的。 | 1 | 2 | 3 | 4 | 5 |
| 1. 您会推荐您的社区全科医生给您的家人和朋友。 | 1 | 2 | 3 | 4 | 5 |
| 综合医院专科医生方面 | | | | | |
| 1. 您对您的综合医院医生的专业能力很自信。 | 1 | 2 | 3 | 4 | 5 |
| 1. 您相信您在综合医院医生给您提供的服务。 | 1 | 2 | 3 | 4 | 5 |
| 1. 您乐意与您的综合医院医生讨论关于您的疑虑和健康问题。 | 1 | 2 | 3 | 4 | 5 |
| 1. 您的综合医院医生理解您对他/她就您的健康问题的表述。 | 1 | 2 | 3 | 4 | 5 |
| 1. 您的综合医院医生给予您的信息容易理解。 | 1 | 2 | 3 | 4 | 5 |
| 1. 您的综合医院医生给予您的信息是有效的。 | 1 | 2 | 3 | 4 | 5 |
| 1. 您会推荐您的综合医院医生给您的家人和朋友。 | 1 | 2 | 3 | 4 | 5 |
| 转诊及其他方面： | | | | | |
| 1. 您相信参与您的医疗行为的人员都了解您以前的病史。 | 1 | 2 | 3 | 4 | 5 |
| 1. 您的社区全科医生在您向他/她解释前知道综合医院医生给予您的建议。 | 1 | 2 | 3 | 4 | 5 |
| 1. 您的综合医院医生在您向他/她解释前知道社区全科医生给予您的建议。 | 1 | 2 | 3 | 4 | 5 |
| 1. 您的社区全科医生同意您在综合医院医生的建议 | 1 | 2 | 3 | 4 | 5 |
| 1. 您的社区全科医生和您的综合医院医生有很好的沟通。 | 1 | 2 | 3 | 4 | 5 |
| 1. 您的综合医院医生经常同意您在社区全科医生的建议。 | 1 | 2 | 3 | 4 | 5 |
| 1. 您认为您的诊疗信息在社区机构和综合医院间互通很重要。 | 1 | 2 | 3 | 4 | 5 |
| 1. 您在综合医院接受服务后总能将您转给社区全科医师进行跟进计划。 | 1 | 2 | 3 | 4 | 5 |
| 1. 您坚信您从在综合医院和社区机构得到的服务是协调的。 | 1 | 2 | 3 | 4 | 5 |
| 1. 预约综合医院的医师必须由基层服务中心进行。 | 1 | 2 | 3 | 4 | 5 |
| 1. 您认为由同一个医生（或医疗团队）比同一个医院为你提供服务重要。 | 1 | 2 | 3 | 4 | 5 |
| 1. 您认为由您自由选择服务比通过有效地协调机制为您提供合适的服务重要。 | 1 | 2 | 3 | 4 | 5 |

您的基础资料：

性别： 1、女 2、男 3、不详

年龄（岁）： 1、35以下 2、35—44 3、45—54 4、55—64 5、65以上

文化程度： 1、大专以下 2、大专 3、本科 4、硕士及以上

医疗类型： 1、自费 2、新农合 3、医疗保险 4、公费医疗 5、商业保险

收入水平（元）： 1、2000以下 2、2001—4000 3、4001—6000

4、6001—8000 5、8001—10000 6、10000以上

No： ________

Survey on consumer perspective of continuity of health care

**Instructions:** These questions were designed to gather information about your opinion and experience on continuity of health care. Please feel free to express your own thoughts. Your contribution to this study may help us to develop relevant policies in the future.

Please read each question carefully, and choose one of the numbers (ranging from 1 to 5) to reflect your opinion and situation. Please not 1 indicates “strongly disagree”, 2 indicates “disagree”, 3 indicates “neutral”, 4 indicates “agree”, and 5 indicates “strongly agree”.

| Whether you agree | Strongly disagree | disagree | Neutral | Agree | Strongly agree |
| --- | --- | --- | --- | --- | --- |
| About general practitioners in the community health services | | | | | |
| 1. I have confidence in the professional ability of my GP | 1 | 2 | 3 | 4 | 5 |
| 1. I believe that my GP cares about me | 1 | 2 | 3 | 4 | 5 |
| 1. I feel comfortable consulting my GP about my   doubts or health problems | 1 | 2 | 3 | 4 | 5 |
| 1. My GP understands what I tell him/her about my   health | 1 | 2 | 3 | 4 | 5 |
| 1. The information my GP gives me is easy to understand | 1 | 2 | 3 | 4 | 5 |
| 1. The information my GP gives me is sufficient | 1 | 2 | 3 | 4 | 5 |
| 1. I would recommend my GP to my family and friends | 1 | 2 | 3 | 4 | 5 |
| About specialists in the hospitals | | | | | |
| 1. I have confidence in the professional ability of the specialists treating me | 1 | 2 | 3 | 4 | 5 |
| 1. I believe that the specialists care about me | 1 | 2 | 3 | 4 | 5 |
| 1. I feel comfortable consulting the specialists about my doubts | 1 | 2 | 3 | 4 | 5 |
| 1. The specialists understand what I tell them about my   health | 1 | 2 | 3 | 4 | 5 |
| 1. The information the specialists give me is easy to understand | 1 | 2 | 3 | 4 | 5 |
| 1. The information the specialists give me is sufficient | 1 | 2 | 3 | 4 | 5 |
| 1. I would recommend my specialists to my family   and friends | 1 | 2 | 3 | 4 | 5 |
| Referral and care coherence | | | | | |
| 1. I believe that the professionals attending to me know my previous medical history | 1 | 2 | 3 | 4 | 5 |
| 1. My GP is aware of the instructions given to me by the specialist before I explain them to him/her | 1 | 2 | 3 | 4 | 5 |
| 1. The specialist is aware of the instructions given to me by my GP before I explain them to him/her | 1 | 2 | 3 | 4 | 5 |
| 1. My GP is in agreement with the specialist’s instructions | 1 | 2 | 3 | 4 | 5 |
| 1. My GP and my specialist communicate with each other | 1 | 2 | 3 | 4 | 5 |
| 1. The specialist is usually in agreement with my GP’s instructions | 1 | 2 | 3 | 4 | 5 |
| 1. It is important to share information between community and hospital facilities | 1 | 2 | 3 | 4 | 5 |
| 1. The specialist sends me to my GP for follow-ups | 1 | 2 | 3 | 4 | 5 |
| 1. I believe that the care I receive from my GP and the specialist is coordinated | 1 | 2 | 3 | 4 | 5 |
| 1. Hospital appointments must be arranged by community facilities | 1 | 2 | 3 | 4 | 5 |
| 1. It is more important to have access to the same doctor (or a team) than to the same facility (hospital) | 1 | 2 | 3 | 4 | 5 |
| 1. It is more important to have freedom of choice than to have access to coordinated services through a health facility | 1 | 2 | 3 | 4 | 5 |

Basic information about you:

| Sex: | 1. Female  2. Male  3. Other |
| --- | --- |
| Age (years): | 1. <35  2. 35-44  3. 45-54  4. 55-64  5. 65 and older |
| Education: | 1. Below associate degree  2. Associate degree  3. Bachelor degree  4. Postgraduate qualification |
| Health insurance: | 1. No insurance  2. New Rural Medical Cooperation Scheme  3. Urban health insurance  4. Free medicine  5. Commercial insurance |
| Monthly income (Chinese Yuan): | 1. <2000  2. 2001-4000  3. 4001-6000  4. 6001-8000  5. 8001-10000  6. >10000 |
