# Supplementary material for: Relationship preferences and experience of primary care patients in continuity of care: a case study in Beijing, China
Source: BMC Health Serv Res. 2017 Aug 22;17:585. doi: 10.1186/s12913-017-2536-1 (PMC5568350; doi:10.1186/s12913-017-2536-1)
Supplement: Supplementary file 2 — CFA Results. (DOCX 135 kb) [file 12913_2017_2536_MOESM2_ESM.docx]

**Annex: Goodness-of-fit of model: Confirmatory Factor Analysis (CFA)** **of the modified Questionnaire of Continuity between Care Levels (CCAENA)**
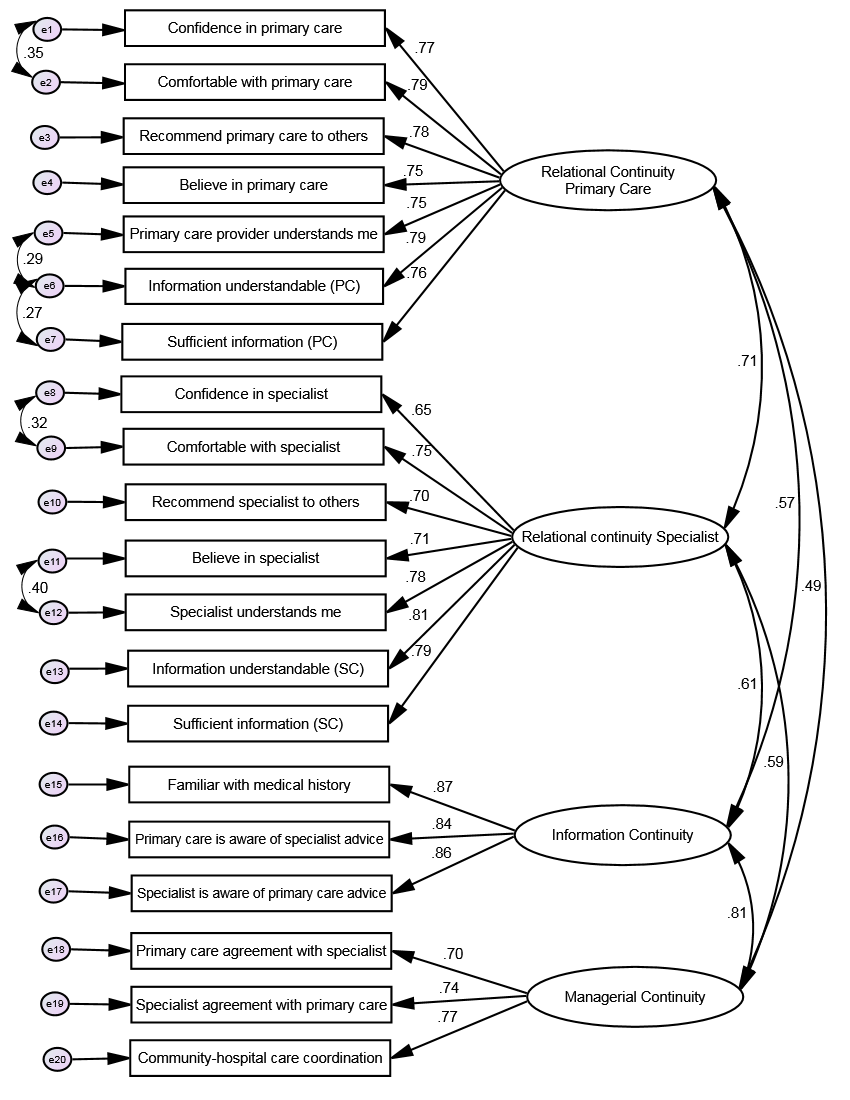


Note: The model allows correlation of errors with high covariation within a domain. GFI=0.912; CFI=0.943; NFI=0.927; TLI=0.932; RMSEA=0.069 (90% CI 0.063, 0.074); χ^2^(df)=683.358 (159), p<0.001.
